# Exploiting Data Distribution: A Multi-Ranking Approach

**DOI:** 10.3390/e27030278

**Published:** 2025-03-07

**Authors:** Beata Zielosko, Kamil Jabloński, Anton Dmytrenko

**Affiliations:** Institute of Computer Science, University of Silesia in Katowice, Bȩdzińska 39, 41-200 Sosnowiec, Poland; kjablonski1@us.edu.pl (K.J.); tonydnipro@gmail.com (A.D.)

**Keywords:** feature selection, ranking of attributes, decision trees, decision rules, greedy algorithm, ensemble, distributed data

## Abstract

Data heterogeneity is the result of increasing data volumes, technological advances, and growing business requirements in the IT environment. It means that data comes from different sources, may be dispersed in terms of location, and may be stored in different structures and formats. As a result, the management of distributed data requires special integration and analysis techniques to ensure coherent processing and a global view. Distributed learning systems often use entropy-based measures to assess the quality of local data and its impact on the global model. One important aspect of data processing is feature selection. This paper proposes a research methodology for multi-level attribute ranking construction for distributed data. The research was conducted on a publicly available dataset from the UCI Machine Learning Repository. In order to disperse the data, a table division into subtables was applied using reducts, which is a very well-known method from the rough sets theory. So-called local rankings were constructed for local data sources using an approach based on machine learning models, i.e., the greedy algorithm for the induction of decision rules. Two types of classifiers relating to explicit and implicit knowledge representation, i.e., gradient boosting and neural networks, were used to verify the research methodology. Extensive experiments, comparisons, and analysis of the obtained results show the merit of the proposed approach.

## 1. Introduction

Technological advances and the global nature of the activities of many companies and institutions necessitate data processing in a distributed form. This applies not only to the location but also to the nature of the data, which may cover different parts of the company’s operations. Moreover, modern technologies generate huge amounts of data that are difficult to store and process in one place. Data are generated in multiple locations simultaneously and can take different forms depending on the structures in which it is stored.

A domain that plays an important role in this context is distributed data mining (DDM). It is the process of discovering knowledge or patterns from data stored in different locations, using distributed processing techniques [1,2]. The key features regarding DDM are (i) distributed data sources, which may be related to physical, organizational, or legal constraints; (ii) local processing, i.e., data are processed locally in each of the distributed locations to reduce the need to transmit large datasets over the network; (iii) consolidation of results where the results of local analyses are combined to produce a global picture; and (iv) global analysis, i.e., based on the combined findings, the system performs a final analysis for discovering global patterns or relationships. Examples of applications include distributed e-commerce systems analysing user preferences from different regions, for instance, Netflix’s platform that processes user data from different localisations to deliver personalised services [3,4].

Information theory is also used within DDM. Data that are dispersed are often characterised by different statistical distributions. Information theory, through measures such as entropy, allow us to analyse the diversity of these data and determine how much information is contained in the various data sources [5].

Feature selection plays an important role in the data mining domain, especially in the stages of data preprocessing and analysis. The main aim of this process is to identify, from the available set of features, those that are most relevant and have the greatest impact on the decisions to be taken [6]. The main goals of feature selection include enhancing the predictive performance of models, creating faster and more cost-efficient predictors, and offering deeper insight into the underlying process that generated the data [7,8]. It is important due to the nature of distributed systems and large datasets. Attribute selection methods help to reduce the number of analysed features, simplifying models and speeding up processing [9]. They also reduce the amount of data transferred between nodes, decreasing communication costs. When models are built in different locations, limiting to the most relevant attributes improves the consistency of results between nodes and facilitates the consolidation of results from different nodes [10,11,12]. In the framework of information theory and distributed data, measures such as information gain or mutual information should be mentioned to select locally relevant attributes in local sources and globally relevant features in the whole system.

Feature selection can be approached in two main ways. The first group of methods involves ranking features based on a specific criterion and selecting the top *k* features. The second one focuses on identifying the smallest subset of features that maintains the performance of the learning model. In the case of ranking methods, each attribute is assigned a weight to reflect its relevance; then, attributes are sorted, usually in order from most to least relevant, and the top-ranked attributes are used in the analysis. Feature ranking methods employ various metrics, such as similarity scores, statistical measures, information-theoretic approaches, or functions derived from the outputs of classifiers [13,14]. These techniques aim to prioritize features based on their relevance or contribution to a given model, helping to improve interpretability, reduce dimensionality, and enhance the overall performance of the learning algorithm [15,16,17].

The motivation for the proposed approach is the need to discover knowledge and global patterns from distributed data. Processing and analysing such sets is much more difficult than in the case of centralized data. A popular field that is being developed in this context is federated learning, which aims to create a global classification model taking into account the parameters and results of local models. It can be said that federated learning enables collaborative training of machine learning models by sharing models’ updates with a central server for aggregation [18]. This work does not use the federated learning technique to construct a global classifier; however, the goal is to create a global ranking of attributes based on local data sources and obtain from them local rankings. Weights of attributes and their ordering at the intermediate and global levels can be considered a source of knowledge about the most important features in a distributed data environment. Examples of applications include systems that analyse user preferences for a specific problem at the level of individual regions and then at the country level. Another application is systems aimed at supporting resource management, which allow for more effective management of data, processes, and allocation of these resources in distributed environments. For example, attributes with a higher position in the ranking can be treated as corresponding to the key tasks for the operation of the system; therefore, resources such as computing power, memory, and network bandwidth are allocated to them first.

The main contribution of this paper is a research methodology for creating a global ranking of attributes in a distributed environment. Taking into account a knowledge representation perspective, the proposed rankings are created using decision rules. In [19] weighting of attributes based on the greedy algorithm was considered, but only at the local level, and it was applied in the stylometry domain. In this work, the application of such ranking in the hierarchical approach for global ranking construction was proposed. The distributed data environment was obtained using reducts construction as a popular feature selection method in the framework of rough set theory [20,21]. In the proposed methodology, reducts were used as a method to obtain different (in terms of attributes) subsets of the dataset, which are considered as local data sources. Experiments were performed on the dataset related to predicting students’ dropout and academic success issues from the UCI ML Repository [22]. The analysis of the classification accuracy and comparison at the different stages of the global ranking construction is included. Informativeness of features, which consists of attribute rankings, was also studied. In this direction of our research, it is the first time that the verification of local rankings derived from the greedy algorithm for decision rule induction (and global ranking) has been carried out using classification models that are not based on decision rules. This process did not use decision rules filtering but sequentially constructed subtables based on the studied attributes from the ranking. The contribution contains a proposed methodology for the development of a global attribute ranking using the greedy algorithm for the induction of decision rules and the verification of this approach based on classifiers related to decision trees and neural networks.

The methodology was verified through extensive experiments. The dataset was divided into *k* sets, k=1,…,9, each of which included *i* subtables, i=2,…,10. The subtables were obtained by the selection of attributes driven by induced reducts from the entire dataset. For each subtable in the set *k*, local rankings were obtained by using the greedy algorithm for decision rules induction [23]. Then, for each set *k*, a strategy for intermediate rankings construction was proposed, taking into account the properties of the greedy algorithm and the characteristics of the retrieved local rankings. Then, global weights of attributes were obtained. All rankings at the intermediate and global levels were verified from the point-of-view of classification, i.e., the gradient boosting approach [24] and neural network in the form of MLP (multi-layer perceptron) [25]. The entropy of features, which consists of rankings, was also calculated. The constructed classifiers were controlled by the attributes’ ranking positions based on a backward elimination approach. The results of the experiments were validated against the test part of the entire dataset and analysed and compared for intermediate and global levels of ranking construction.

The paper consists of six sections. Section 2 presents the research background related to feature selection and employed methods. Section 3 describes the framework of the proposed research methodology. Experimental results are presented in Section 4. Section 5 includes a comparison and analysis of findings. Conclusions and future research directions are provided in Section 6.

## 2. Background

In this section, information related to feature selection, ranking construction, and selected classifiers is presented.

### 2.1. Feature Selection

The aim of feature selection is to remove irrelevant or redundant attributes [26,27] from the set of available features. Selecting relevant attributes allows the model to focus on the most influential features, leading to improved model efficiency and accuracy. Removing irrelevant variables reduces the risk of “noise” in the data, resulting in more accurate predictions. With large datasets, processing all features can be costly in terms of time and computing resources. Feature selection helps to reduce the number of variables, which reduces model training time and computing power requirements. Models learning on datasets with many features are more prone to overfitting, i.e., adjusting the training data in too much detail. By reducing the number of features, the model is generalized better on new data, improving its predictive ability. The number of features also plays an important role from the point of view of knowledge representation [28,29,30]. Models with fewer features are more comprehensible, which is particularly important in fields such as medicine. A smaller number of features makes it easier to interpret and understand how a model works.

This approach coincides with information theory, which provides mathematical tools for assessing which features in the data are most relevant for predicting the target variable [31,32]. Examples include mutual information (MI), entropy, information gain (IG), and minimal redundancy maximal relevance (mRMR) methods. MI measures how much information about one variable (e.g., target variable Y) is provided by another variable (e.g., feature X) and identifies the attributes most related to predicting the target variable. Entropy measures uncertainty or “heterogeneity” in the data and features with high entropy contain more potential information. Information gain measures how much uncertainty (entropy) of the target variable Y has been removed by including a feature X. It is widely used in decision tree construction algorithms, such as ID3, C4.5. Methods related to mRMR balances maximising the relevance of features relative to the target variable and minimising redundancy between attributes.

Feature selection methods can be divided into three main categories: filter, wrapper, and embedded [33]. Filtering methods assess the importance of features independently of the machine learning model. Variables are selected or discarded before the model training process, which makes these methods considered fast and scalable. Attributes can be selected based on statistical properties, e.g., the Pearson correlation coefficient. Filtering methods often evaluate features individually without taking their interactions into account. This means that features that may be relevant in combination with others may be omitted. As filtering methods do not directly consider the impact of features on the final accuracy of the model, they may not always lead to an optimal set of features in terms of the model.

Wrapper methods select a subset of features based on their impact on the accuracy of a particular predictive model. The selection process is iterative and involves training the model with different subsets of features to assess which features optimize model performance. Feature selection in wrapper methods is closely linked to the model, meaning that features selected in this way maximize the performance of a given algorithm. This leads to better performance than filter methods, especially for complex data with non-linear relationships. However, there is a risk that the set of features may be overfitted to the training set, which will worsen the model’s ability to generalize. If a different algorithm is chosen, feature selection may need to be performed again. Wrapper methods are used when model accuracy is a priority and computational resources are not a significant constraint.

Embedded methods are feature selection algorithms that are integrated into the model learning process. In contrast to filter techniques, embedded methods perform feature selection directly while training the model. These methods are popular in situations where computational efficiency is important and models need to be optimized for accuracy while maintaining simplicity. Examples are decision trees and their extensions, e.g., Random Forest, Gradient Boosting, or SVM methods, with appropriate modifications.

### 2.2. Ranking Construction

Attribute rankings are methods of ordering features (assigning weights) according to their importance in a given context, for example, machine learning models. The aim is to identify which attributes have the greatest impact on the outcome or are most important in predicting a given variable [14].

There are many approaches to assessing and ranking attributes [6,34,35,36], which can be divided into several main categories: (i) statistically based methods that use correlation, variance, and others measures to analyse the significance of features based on their distribution; (ii) machine learning-based methods that use, e.g., decision trees as Random Forest; (iii) feature selection-based methods, e.g., Relief algorithm or approach based on reducts; and (iv) information theory-based methods that use, for example, entropy or information gain.

In this work, we will use a decision rules-based approach for weighting attributes at the local level, i.e., for each subtable in the set *k*. Local rankings are obtained based on the greedy algorithm, which is known for its application to the set cover problem [37]. The motivation for selecting this algorithm is the issue of knowledge representation in the intuitive form of decision rules. Based on previous studies [23], it has been proven that this algorithm allows for the construction of short decision rules and, by making certain assumptions on the class NP, this algorithm allows for obtaining results close to those obtained by the best polynomial approximate algorithms. Short decision rules are easy to understand and interpret. In addition, the length of the rules, i.e., the number of descriptors (attribute = value pairs) forming the premises of the rule, is an important indicator of the quality of the rule. Another popular measure of rule quality is support, which is the number of objects in the dataset for which the left and right sides of the rule are met. Rules with high support allow the discovery of relevant patterns from the data.

An important property of the greedy algorithm is that the attributes forming the rule are characterised by the high separability of objects from other decision classes. This is due to the nature of this algorithm, which, in each iteration during the process of rule construction, selects an attribute that separates a maximum number of rows with a different decision. This algorithm works sequentially for each row row of the dataset represented by table *T*. U(T,row) denotes a set of rows from *T* that are labeled with a class label different from class *d* attached to the considered row. The Algorithm 1 presents the pseudocode of the greedy algorithm for the construction of the decision rule for row of *T*.
**Algorithm 1** Greedy algorithm for the construction of decision rules**Require:** Dataset *T* with attributes $a_{1},\ldots ,a_{m}$, row $row=(b_{1},\ldots ,b_{m})$ of *T* labeled by *d*.**Ensure:** Decision rule for row of *T*.Q⟵∅;**while** attributes from *Q* separate from row less than U(T,row) rows **do**   select $a_{i}\in \left\{a_{1},\ldots ,a_{m}\right\}$ with minimal index *i* such that $a_{i}$ separates from row the   maximal number of rows unseparated by attributes from *Q*   $Q⟵Q\cup \{a_{i}\}$.**end while**${⋀}_{a_{i}\in Q}\left(a_{i}=b_{i}\right)\to d$.

In the presented approach, local rankings were constructed taking into account: (i) the number of rules in which the given attribute exists, (ii) the number of rows separated by the attribute, and (iii) the maximum support of the rule including the considered variable.

The construction of rankings at higher levels takes into account the number of rankings in which the attribute appears. If these values for two or more attributes are the same, the highest weight of the attribute at the lower ranking level is taken into account.

In the paper, for rankings created at higher levels, i.e., the so-called intermediate and global levels, entropy was calculated. This measure, determined for a set of attributes, assesses how much information (or uncertainty) these features contain. A high value indicates that the attribute (or set of features) is more diverse and potentially more informative [38]. In the context of data analysis, the entropy of a set of features $A=\{a_{1},\ldots ,a_{n}\}$ is defined by the formula:

(1) $$H\left(A\right)=-\sum_{i=1}^{n}p\left(a_{i}\right)\log_{2}p\left(a_{i}\right),$$

where $p\left(a_{i}\right)=\frac{w_{i}}{\sum_{j=1}^{n}w_{j}}$, and $w_{i}$ is a weight assigned to a given attribute. The maximum entropy $H_{max}$ for the n-elements set is $\log_{2}n$, and it is achieved assuming that all elements have equal probability $\frac{1}{n}$. The small difference $H_{max}-H\left(A\right)$ suggests that the features are highly informative.

### 2.3. Selected Classifiers

Two different types of classifiers were used to verify the proposed research procedure: gradient boosting and neural networks.

Gradient Boosting is one of the most popular and efficient machine learning techniques, particularly used in regression and classification tasks [39]. The method is based on the idea of iteratively building an ensemble of models in the form of decision trees in such a way that each successive model corrects the errors of its predecessor. Thus, gradient boosting achieves high accuracy and is widely used in the field of data analysis and artificial intelligence. The cost function in gradient boosting is a very important element that determines how the model learns from the data. Log-loss and cross-entropy are the most popular in classification due to their ability to handle probabilistic predictions and penalise errors in a way that is proportional to the confidence of the predictions [40]. Logarithmic Loss (Log-Loss) is the most commonly used cost function for binary and multi-class classification tasks [41]. The name of the “gradient boosting” method derives from the use of the gradient—the direction of greatest decrease in the cost function—for optimisation [42]. In the research performed, the method was implemented using the XGBoost (ang. eXtreme Gradient Boosting) library.

The second classifier used in the verification of the proposed methodology is based on implicit knowledge representation, i.e., the neural network in the form of multi-layered perceptron, or MLP [43]. It is a versatile supervised learning algorithm used for classification and regression tasks. It is constructed of multiple interconnected layers of artificial neurons, where each layer transforms the input data through non-linear activation functions, which can be selected by the operator manually. MLPs are trained by iteratively adjusting the weights between neurons to minimize a loss function, effectively learning the underlying patterns within the data for classification purposes. The loss function can be both preset and selected automatically based on the operator’s needs.

## 3. Framework of Multi-Ranking Construction

In this section, detailed descriptions of all steps of the proposed research methodology and performed tasks are presented.

### 3.1. Framework of Developed Methodology

The research conducted within the framework of the proposed approach includes the following steps:

- Data preparation;
- Induction of reducts;
- Construction of sets of subtables and data scattering;
- Local rankings construction based on the decision rules induced by the greedy algorithm for each set *k*, k=1,…,9, with *i* subtables, i=2,…,10.
- Intermediate ranking construction, for each set *k*;
- Global ranking construction;
- Verification of the importance of attributes at the intermediate and global levels using a backward elimination approach driven by the attribute’s ranking position and using gradient boosting and multi-layer perceptron methods;
- Analysis and comparison of obtained results.

**Figure 1:**
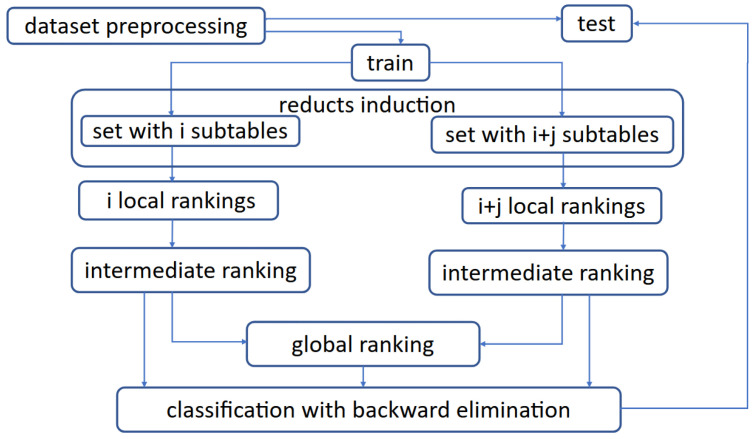
Framework of developed methodology.

The Figure 1 presents a general overview of the developed methodology applied to distributed data.

### 3.2. Data Description and Preparation

The Predict Students Dropout and Academic Success dataset [22] consists of anonymised data collected from a higher education institution, i.e., Polytechnic University of Portalegre, Portugal. It is designed to predict student dropout risk and academic performance based on various attributes of students. The dataset includes 37 features, including a class label, both categorical and numerical, representing various factors such as (i) demographics (for example, age, gender, nationality), (ii) academic information (for example, grades, GPA, education), and (iii) socio-economic factors (as scholarship holder, tuition fees up to date, parental education level, and others). There are three values for the class label: “Dropout”, “Enrolled”, and “Graduate”, which refer to the student’s status at the end of the normal term. The dataset consists of 4424 rows, and for the purpose of experiments and validation of the proposed approach, it was divided in the proportions 70% training part and 30% testing part. Table 1 presents information about attributes included in the dataset, i.e, column Id contains the code of the attribute (which will be used later), and column Attribute contains the name of the attribute.

For the five attributes in the set, a discretisation of their values was carried out, that is: a7—previous qualification (grade), a13—admission grade, a20—age at enrollment, a26—curricular units 1st sem (grade) and a32—curricular units 2nd sem (grade). For this purpose, the Fayad and Iranii algorithm [44] was used as a supervised discretisation method with default settings available in the WEKA software [45].

### 3.3. Reducts and Data Distribution

In rough set theory, reducts are a popular feature selection method belonging to the group of filter category algorithms. There are different types of reducts, different definitions depending on the adopted criteria, and different algorithms for reduct construction [23,26]. From a classification perspective, a reduct is a minimal subset of attributes that has the same power to distinguish objects with different class labels as the full set of attributes. A reduct can also be defined as a minimal set of attributes that preserves the degree of dependency on the full set of attributes. The problem of finding different versions of reducts in data is NP-hard [37], so heuristic approaches are often used.

In the paper, reducts were constructed by using the genetic algorithm implemented in the RSES (i.e., Rough Set Exploration System) [46]. This algorithm enables the construction of a sufficiently large number of reducts within a reasonable timeframe [47]. It utilizes a binary genetic algorithm, incorporating traditional binary operators such as mutation and crossover, along with the “roulette wheel” selection method. The computation process has been optimized using an additional structure known as the “discernibility matrix” [48]. This is a binary matrix where each column represents an attribute and each row corresponds to a pair of distinct objects. If an attribute has different values for a pair of objects, a value of 1 is placed at the intersection of the corresponding column and row. Finding a reduct involves identifying the smallest subset of columns that covers the entire matrix.

In the framework of the performed experiments, for the considered dataset, 100 reducts were induced by the genetic algorithm. From this set, 54 were selected for the construction of subtables and divided into *k* sets, k=1,…,9. Table 2 presents the characteristics of the obtained reducts, i.e, their lengths per set *k*. All reducts generated by the genetic algorithm contain between 10 and 13 attributes, which represent a relatively large percentage reduction in the number of attributes concerning the full set of features.

Table 3 presents all attributes included in reducts from the set k=9. For all ten reducts, the first three attributes are the same, and then the number of different attributes increases with the length of the reduct.

Based on the reducts from the input data table, subtables were created in such a way that each subtable contains only the columns corresponding to the attributes present in the given reduct. Table 4 describes characteristics, i.e., the number of rows after removing duplicates (row rows) and the number of columns (row attr) for the obtained subtables, for sets *k*, k=1,…,9. Columns of Table 4 are labeled by numbers from 2 to 10, which correspond to the number of subtables in the set *k*. These are average values.

In the subtables created, the number of columns corresponds to the cardinalities of reducts presented in Table 2. The number of rows corresponds to the training part of the dataset and is about 70% of the rows from the entire dataset.

### 3.4. Importance of Attributes at Intermediate and Global Levels

For all 54 subtatables, local rankings based on decision rules induced by the greedy algorithm were constructed. For each subtable, a set of unique decision rules was induced, and then the weights of attributes were calculated, taking into account the number of rules in which the attribute appears $w_{a,1}$. If two or more attributes have the same value, then the number of separated rows with different class labels was calculated as a ratio of rows separated by the given attribute-value pair and decision class to the total number of objects in the set with a different decision $w_{a,2}$. An attribute with a higher ratio has been assigned a higher rank. If there were attributes with the same ratio values, then the maximum rule support among the rules for which the ratio of separated rows is maximal was taken into account $w_{a,3}$.

Considering the way the data are distributed, i.e., into *k* sets, each containing *i* subtables, higher-level rankings, called intermediate rankings RI, were constructed based on local rankings. Each intermediate ranking is assigned to the set *k*, and they were created using *i* local rankings. Both intermediate and global level rankings follow the principle of the number of occurrences of attributes in the lower-level rankings.

Let $WL_{a}^{\left(n\right)}$ be a weight of attribute *a* in the local ranking *n* represented as a vector:

(2) $$WL_{a}^{\left(n\right)}=\left[\begin{array}{ccc} w_{a,1}^{\left(n\right)} & w_{a,2}^{\left(n\right)} & w_{a,3}^{\left(n\right)} \end{array}\right].$$

For *i* local rankings in the set *k*, the weight $WI_{a}^{\left(k\right)}$ of attribute *a* in the intermediate ranking *k* is defined as:

(3) $$WI_{a}^{\left(k\right)}=\max_{n=1}^{i}WL_{a_{j}}^{\left(n\right)},j=1,2,3.$$

Let $w_{a,4}^{\left(k\right)}$ denote the number occurrence of a given attribute *a* in the set *k* with *i* local rankings:

(4) $$w_{a,4}^{\left(k\right)}=\sum_{n=1}^{i}1\left(a\in WL_{a}^{\left(n\right)}\right),$$

where $1(a\in WL_{a})$ is an indicator function that equals 1 if attribute *a* is present in the given ranking and 0 otherwise.

Similarly, for *k* intermediate rankings, the global weight $WG_{a}$ of attribute *a* in the global ranking is defined as:

(5) $$WG_{a}=\max_{s=1}^{k}WI_{a_{j}}^{\left(s\right)},j=1,2,3,$$

and respectively

(6) $$w_{a,4}^{\left(G\right)}=\sum_{s=1}^{k}1\left(a\in WI_{a}^{\left(s\right)}\right).$$

**Figure 2:**
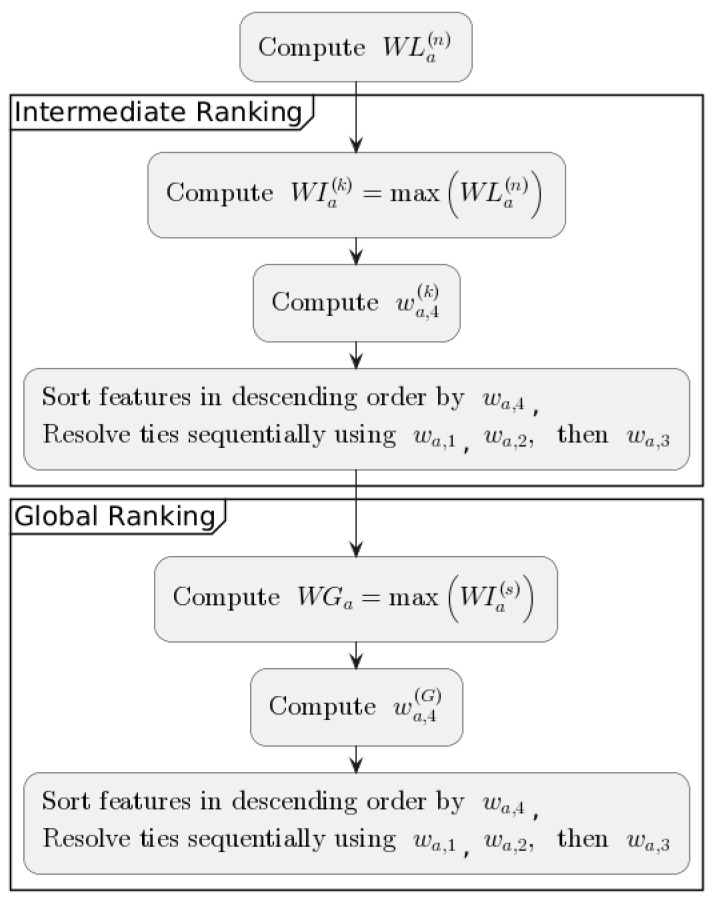
General scheme for rankings construction.

The general scheme for creating rankings is presented in Figure 2.

Table 5 presents the attributes appearing in the intermediate and global rankings. Column Nr indicates the position of the attribute in the ranking. Columns from RI_2 to RI_10 present attributes that consist of intermediate rankings, and the number from 2 to 10 corresponds to the number of local rankings in the set *k*. Column RG denotes the global ranking.

On the basis of the results obtained, it can be seen that three attributes, such as a8—nationality, a15—educational special needs, and a21—international, did not feature in any of the intermediate rankings. The ones presented in Table 5 contain a varying number of attributes, and usually, this number increases as the number of local rankings in set *k* increases. The global ranking contains 33 attributes and among those given, the 10 highest weightings are: course, application mode, inflation rate, mother’s qualification, mother’s occupation, application order, father’s qualification, curricular units 2nd sem (grade), previous qualification (grade), and curricular units 1st sem (grade). The lowest positions are assigned to attributes: admission grade, daytime/evening attendance, debtor, and curricular units 1st sem (without evaluations).

**Figure 3:**
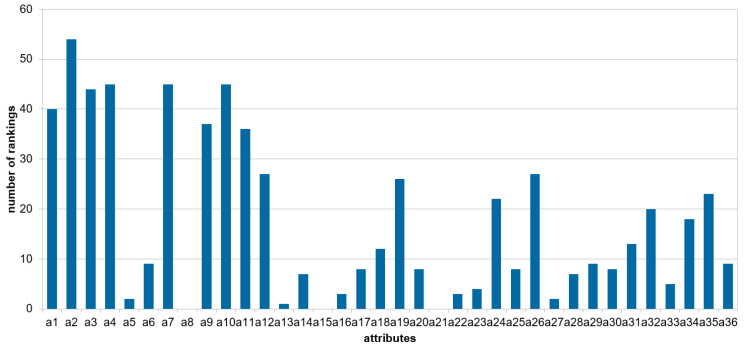
Occurrence of attributes in the local rankings.

Figure 3 presents, for each attribute, the number of local rankings constructed by the greedy algorithm in which the attribute occurs.

It can be seen that the attribute a2 is present in all local rankings, while the attribute a4 is present in 45 rankings out of 54; however, in the global ranking (see Table 5), a4 is in the first position. It should be noted that this figure shows the frequency of occurrence of the attributes in the rankings and not the weights, which determine the order of the attributes, as shown in Table 5 for intermediate and global levels. Attributes a8, a15, and a21 did not appear in any of the local rankings, so they are not presented in Figure 3; they also do not appear in the intermediate and global levels.

Table 6 presents differences between $H_{max}$ and H(A) for intermediate and global rankings.

**Figure 4:**
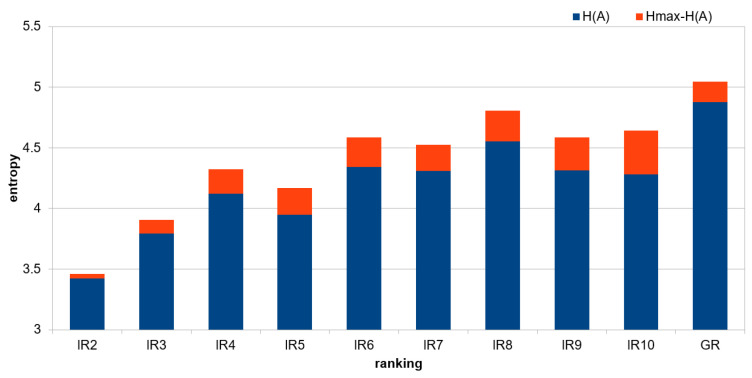
Informativeness of attribute rankings.

This visualisation is also shown in Figure 4.

The biggest difference is visible for RI_10, RI_9, and RI_8, which means that the data in the set are diverse; this may indicate greater informational value. Taking into account the number of local rankings on the basis of which the intermediate rankings were created, it can be observed that as the number of local rankings increases, the value of the entropy difference $H_{max}$ and H(A) usually increases as well, which indicates that the data is structured and informative.

## 4. Experimental Results

This section presents the performance of classifiers obtained based on intermediate and global rankings.

Training sets for the construction of classifiers were created in the form of subtables containing the attributes corresponding to the attributes included in the given ranking at the intermediate and global levels, respectively. For the constructed classification models, a backward elimination technique was applied, driven by the attributes included in a given ranking. Starting with the attribute in the lowest position in the ranking, the number of attributes in the subtable was sequentially reduced by removing them, and the classification accuracy was evaluated on the test set. This process was repeated until the accuracy was relatively low or the attributes were exhausted. To evaluate the effectiveness of a model, the accuracy of classification was used. It indicates the number of correctly classified objects relative to all objects in the test set.

The classification accuracy of a model trained on the dataset containing all attributes from the ranking is treated as a reference point in the local context; the accuracy of the model trained on the dataset containing all 36 attributes is considered the reference point in the global context.

In the research, XGBoost and MLP algorithms were used for the construction of classifiers. Default parameters were used for the XGBoost classifier. The MLP classifier was used with the following parameters: random_state=3, max_iter=1000; the rest of parameters were kept default to sklearn implementation. It is important to note that the number of maximum iterations in this case is the number of iterations the solver stops at if convergence has not been reached earlier.

The computational complexity of the main algorithms, with *m* as the number of attributes and *n* as the number of instances is as follows: the greedy algorithm for the induction of decision rules: $O(m\cdot n^{2})$; XGBoost for the construction of the model: O(n·log(n)m); MLP for the construction of the model: O(m·n).

Table 7 presents reference values in the global context for XGBoost and MLP. In this case, XGBoost allows us to obtain slightly better results than MLP.

Table 8 and Table 9 present the accuracy of classifiers at the intermediate and global levels. Results in Table 8 are related to XGBoost classifiers in Table 9—MLP classifiers. In both tables, column Nr indicates the attribute’s position in the ranking. Columns from RI_2 to RI_10 present accuracy related to intermediate rankings; column RG denotes the global ranking. The values presented in bold indicate the accuracy that is equal to or greater than the reference value in the local context, i.e., the accuracy obtained by taking into account all attributes included in the given ranking.

Among the intermediate rankings, the biggest improvement is seen for the RI_7 ranking, where instead of 23 attributes, 16 attributes are used, achieving a higher accuracy of classification. This number of attributes represents almost 50% of the attributes in the entire dataset. Improvements are also visible for ranking RI_2, RI_3, and RI_6. Considering the global ranking, the highest classification accuracy, surpassing the local reference point, was obtained using only 26 attributes instead of 33. The performance of the classifier at this level is the same as for attribute 26 in ranking RI_8; however, the local reference point was not exceeded in this case. Overall, the obtained results demonstrate a trend where, for attributes occupying the top positions in the rankings, the accuracy of classification generally increases as the number of attributes grows.

Table 9 presents the accuracy of MLP classifiers at the intermediate and global levels.

**Figure 5:**
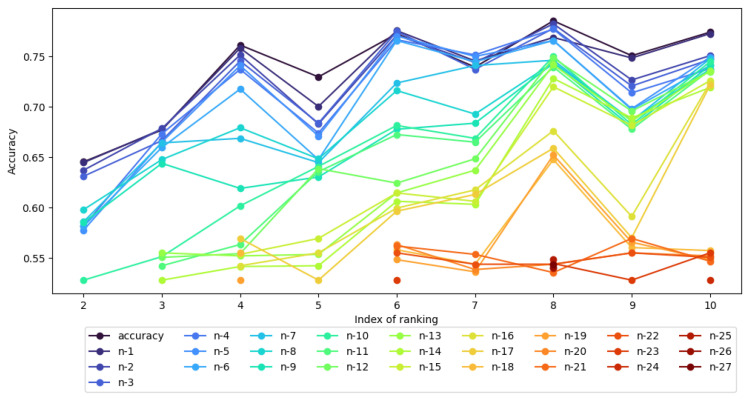
Accuracy of XGBoost on intermediate rankings with $n_{k}-j$ attributes.

**Figure 6:**
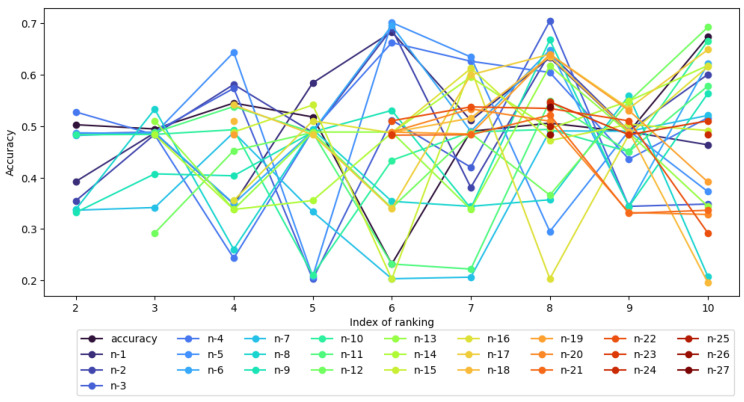
Accuracy of MLP on intermediate rankings with $n_{k}-j$ attributes.

The results in Table 9 are much more varied than in Table 8, as confirmed by the visualizations in Figure 5 and Figure 6. It can be seen that the results obtained for the neural network-based classifier show that for each intermediate and global ranking, an accuracy of classification equal to or greater than the reference point in the local context can be indicated for fewer attributes than the number of attributes in the ranking. In the case of rankings RI_6, RI_7, RI_8, a single attribute in the table is sufficient; however, note the relatively low classification value obtained for the full set of attributes, especially in ranking RI_6. In the case of the global ranking, instead of 33, 15 attributes in the table are sufficient to obtain a classification accuracy above the local reference value, which is 0.575.

Figure 5 shows the accuracy of the XGBoost classifier for each intermediate ranking with *j* last features from the said ranking removed. It can be interpreted as a visual representation of Table 8. Each line and color represent a subset of $n_{k}-j$ attributes in general, where for each intermediate ranking: $n_{k}=\left|RI\_k\right|,k\in \left[2,10\right]$ and $j\in [1,n_{k}-1]$. The line listed as “accuracy” is shows the classifier performance on all attributes from *k*-th ranking, or in other words, “base” performance for each intermediate ranking.

Figure 6 is a form of visual representation of Table 9 and shows the accuracy of the MLP classifier for each intermediate ranking where “accuracy” represents classifier performance on all attributes across each RI_k.

Taking into account the classification accuracy of MLP and XGBoost, the low results obtained by the MLP model are related to the use of a single model, where boosting and the gradient approach were omitted. It should also be noted that in the case of the MLP, the investigation into the provision of hidden layers, nodes, and hyperparameters was omitted, as they are not the subject of this article.

The experiments were conducted using Google Colab, a cloud-based platform that provides a Jupyter Notebook environment with access to GPU and TPU acceleration. The software environment included Python 3.13, along with libraries such as NumPy version 2.1.2, Pandas version 2.2.3, Scikit-learn version 1.5.2, and xgb version 2.1.2.

## 5. Summary of Results

In this section, the best subsets of features for each ranking will be presented for both classifiers, XGBoost and MLP, accordingly. To keep the structure intact, we will start with the results obtained with intermediate rankings, outlining the best performing subsets for each ranking, followed by the performance levels obtained for the global ranking.

Table 10 represents a qualitative improvement (if present) for each intermediate and global ranking with both classifiers used in this research. We can observe a tendency for MLP to severely underperform compared to XGBoost on the full set of features per ranking, leading to smaller subset selections for all rankings. However, only a few reached close to the performance level of XGBoost, which directly points to the advantage of gradient boosting tree-based classifiers when applied to the selected dataset. In this table, acc. denotes reference accuracy in the local context for each $RI_{k}$, $n_{k}-j\;feat.$ denotes the number of last attributes removed from the ranking at which maximum accuracy, denoted as max(acc.), was obtained. The absolute difference in accuracy between acc. and max(acc.) is noted as ▵acc.

**Figure 7:**
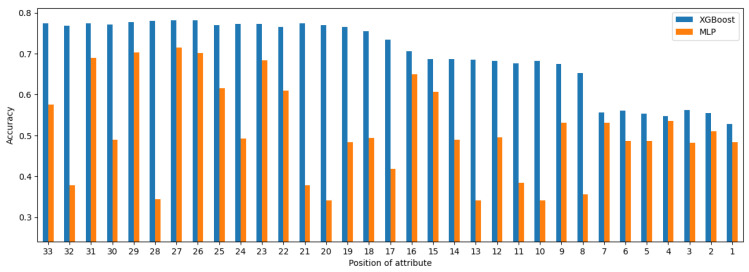
Performance of XGBoost and MPL on RG.

With the context of classifiers and the dataset used, one can observe that only XGBoost yielded measurable improvement in performance both on intermediate and global rankings, with MLP not being able to keep up. This is clearly visible in Figure 7, which shows a side-by-side comparison of the performance of XGBoost and MLP on global ranking across each $n_{k}-j$ subset. In this figure, the notation of $n_{k}-j$ is replaced with the actual position of the attribute in the global ranking RG.

**Figure 8:**
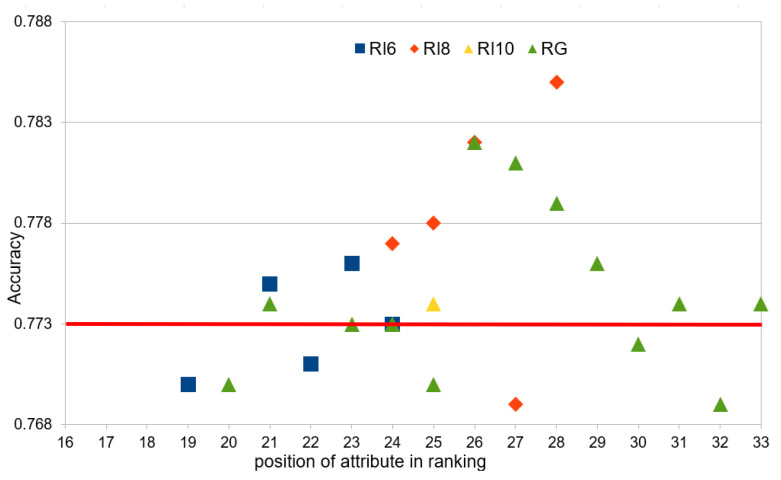
Number of attributes in rankings with higher accuracy than global reference value.

Figure 8 shows the number of attributes in the intermediate rankings and the global rankings that achieved a classification accuracy equal to or greater than the reference point in the global context, i.e., 0.773, in the case of the XGBoost classifier.

In order to obtain a classification accuracy of at least the reference level, 21 attributes, instead of 36, in the rankings RI_6 and RG are sufficient, while the greatest difference from the reference value was obtained in the ranking RI_8, for the whole set of attributes, i.e., for 28 out of 36. Unfortunately, the results obtained for the MLP classifiers are much more divergent than in the case of XGBoost and did not exceed the global reference value.

## 6. Conclusions

In the paper, a research methodology for ranking construction from distributed data was proposed. The main contributions consist of (i) a data distribution approach based on reducts as a feature selection method, (ii) procedures for ranking construction at different levels, i.e., local with 54 rankings, 9 at the intermediate level, and 1 at the global level, and (iii) a method for ranking verification using a backward elimination strategy driven by attributes included in the rankings. Two different types of classifiers were used, related to implicit (MLP) and explicit (XGBoost) knowledge representation.

For the analysed dataset, it was observed that when employing the XGBoost classifier, even the attributes positioned at the lower end of the ranking contribute meaningfully to the construction of the classification model. In contrast, the application of the MLP classifier does not exhibit such a pattern, with the classification results demonstrating greater variability and less consistent reliance on the ranking order of attributes. It should be noted that in many cases, improvements were visible, which allowed for a reduction in the number of attributes and greater classification accuracy than the referenced values at the intermediate and global levels. The extensive experiments performed show the merit of the proposed methodology for multi-ranking construction. In the future, other classifiers with knowledge representation properties will be studied, and other strategies for ranking construction in a framework of dispersed data will be investigated.

## Figures and Tables

**Table 1:** Attributes in dataset related to students dropout and academic success.

Id	Attributes	Id	Attributes
a1	Marital Status	a19	Scholarship holder
a2	Application mode	a20	Age at enrollment
a3	Application order	a21	International
a4	Course	a22	Curricular units 1st sem (credited)
a5	Daytime/evening attendance	a23	Curricular units 1st sem (enrolled)
a6	Previous qualification	a24	Curricular units 1st sem (evaluations)
a7	Previous qualification (grade)	a25	Curricular units 1st sem (approved)
a8	Nationality	a26	Curricular units 1st sem (grade)
a9	Mother’s qualification	a27	Curricular units 1st sem (without evaluations)
a10	Father’s qualification	a28	Curricular units 2nd sem (credited)
a11	Mother’s occupation	a29	Curricular units 2nd sem (enrolled)
a12	Father’s occupation	a30	Curricular units 2nd sem (evaluations)
a13	Admission grade	a31	Curricular units 2nd sem (approved)
a14	Displaced	a32	Curricular units 2nd sem (grade)
a15	Educational special needs	a33	Curricular units 2nd sem (without evaluations)
a16	Debtor	a34	Unemployment rate
a17	Tuition fees up to date	a35	Inflation rate
a18	Gender	a36	GDP

**Table 2:** Cardinalities of reducts.

Set *k*	Length of Reducts									
1	10	10								
2	11	12	12							
3	15	11	11	11						
4	12	12	12	12	11					
5	12	12	12	15	10	10				
6	10	11	11	11	12	13	13			
7	14	12	12	12	12	13	12	12		
8	11	11	11	11	11	12	12	12	12	
9	12	12	12	12	13	10	10	10	10	10

**Table 3:** Attributes included in reducts from the set k=9.

	1	2	3	4	5	6	7	8	9	10	11	12	13
reduct1	a2	a3	a4	a6	a7	a10	a12	a18	a19	a28	a30	a36	
reduct2	a2	a3	a4	a6	a7	a10	a12	a18	a19	a28	a30	a34	
reduct3	a2	a3	a4	a6	a7	a10	a12	a17	a19	a25	a29	a36	
reduct4	a2	a3	a4	a7	a9	a10	a11	a12	a18	a26	a31	a33	
reduct5	a2	a3	a4	a7	a9	a10	a11	a14	a19	a22	a26	a33	a35
reduct6	a2	a3	a4	a7	a9	a10	a11	a31	a32	a35			
reduct7	a2	a3	a4	a7	a9	a10	a11	a26	a31	a35			
reduct8	a2	a3	a4	a7	a9	a10	a11	a30	a31	a34			
reduct9	a2	a3	a4	a7	a9	a12	a19	a30	a31	a36			
reduct10	a2	a3	a4	a7	a9	a12	a19	a30	a31	a34			

**Table 4:** Characteristics of distributed subtables.

	2	3	4	5	6	7	8	9	10
rows	3074.0	3086.7	3079.2	3086.2	3079.5	3075.7	3075.8	3081.7	3077.6
attr	10.0	11.7	12.0	11.8	11.8	11.6	12.4	11.4	11.1

**Table 5:** Intermediate and global rankings of attributes for distributed data.

Nr	RI_2	RI_3	RI_4	RI_5	RI_6	RI_7	RI_8	RI_9	RI_10	RG
1	a4	a4	a4	a4	a4	a2	a2	a4	a4	a4
2	a24	a2	a2	a2	a2	a3	a1	a2	a2	a2
3	a2	a3	a9	a9	a10	a10	a11	a12	a3	a35
4	a9	a9	a3	a3	a3	a1	a34	a7	a7	a9
5	a3	a11	a7	a7	a7	a11	a10	a11	a10	a11
6	a11	a32	a1	a26	a1	a26	a7	a10	a9	a3
7	a10	a10	a35	a1	a24	a12	a9	a1	a12	a10
8	a26	a7	a10	a24	a11	a9	a3	a24	a31	a32
9	a1	a1	a19	a10	a19	a7	a32	a32	a19	a7
10	a34	a35	a24	a19	a12	a4	a12	a26	a30	a26
11	a35	a18	a11	a35	a35	a24	a20	a35	a11	a1
12		a36	a32	a34	a9	a35	a4	a9	a35	a12
13		a23	a26	a28	a26	a36	a31	a14	a36	a24
14		a22	a25	a12	a30	a29	a29	a34	a34	a34
15		a6	a31	a11	a34	a19	a6	a3	a26	a19
16			a12	a32	a17	a31	a19	a19	a6	a25
17			a27	a29	a16	a25	a18	a20	a18	a17
18			a17	a17	a31	a32	a25	a18	a28	a31
19			a16		a25	a28	a24	a6	a33	a36
20			a14		a32	a5	a23	a25	a25	a29
21					a29	a34	a26	a36	a32	a18
22					a27	a23	a17	a22	a22	a6
23					a20	a33	a33	a17	a29	a28
24					a18		a35	a13	a17	a14
25							a30		a14	a30
26							a36			a23
27							a28			a22
28							a14			a33
29										a20
30										a27
31										a16
32										a5
33										a13

**Table 6:** Informativeness of the set of attributes.

RI_2	RI_3	RI_4	RI_5	RI_6	RI_7	RI_8	RI_9	RI_10	RG
0.037	0.115	0.201	0.220	0.245	0.213	0.254	0.272	0.361	0.168

**Table 7:** Performance of classifiers including all attributes in dataset.

Classifier	Accuracy
XGBoost	0.773
MLP	0.728

**Table 8:** Performance of XGBoost classifiers for the intermediate and global rankings of attributes.

Nr	RI_2	RI_3	RI_4	RI_5	RI_6	RI_7	RI_8	RI_9	RI_10	RG
1	0.528	0.528	0.528	0.528	0.528	0.544	0.544	0.528	0.528	0.528
2	0.585	0.555	0.555	0.555	0.555	0.553	0.541	0.555	0.555	0.555
3	0.598	0.550	0.569	0.569	0.562	0.538	0.548	0.569	0.550	0.562
4	0.586	0.542	0.542	0.542	0.563	0.536	0.540	0.555	0.547	0.547
5	0.581	0.551	0.553	0.553	0.548	0.544	0.544	0.566	0.552	0.553
6	0.578	0.644	0.541	0.639	0.558	0.613	0.544	0.560	0.548	0.560
7	0.581	0.648	0.552	0.636	0.596	0.617	0.535	0.570	0.557	0.556
8	0.631	0.664	0.554	0.641	0.599	0.606	0.544	0.591	0.721	0.652
9	0.637	0.660	0.563	0.630	0.614	0.603	0.652	0.681	0.724	0.675
10	**0.645**	0.665	0.602	0.648	0.606	0.637	0.648	0.689	0.726	0.681
11	0.645	0.673	0.619	0.645	0.614	0.648	0.659	0.686	0.719	0.676
12		0.666	0.679	0.648	0.624	0.665	0.676	0.696	0.736	0.682
13		**0.678**	0.669	0.671	0.672	0.669	0.720	0.678	0.735	0.684
14		**0.678**	0.718	0.674	0.681	0.684	0.728	0.682	0.736	0.686
15		0.677	0.741	0.684	0.678	0.693	0.742	0.680	0.745	0.686
16			0.737	0.683	0.716	**0.741**	0.748	0.687	0.741	0.706
17			0.745	0.700	0.724	**0.745**	0.739	0.686	0.737	0.734
18			0.752	0.730	0.766	**0.750**	0.750	0.697	0.748	0.755
19			0.758		0.770	**0.752**	0.743	0.698	0.741	0.765
20			0.761		0.767	0.737	0.744	0.714	0.749	0.770
21					**0.775**	**0.744**	0.746	0.721	0.738	0.774
22					0.771	**0.745**	0.766	0.727	0.747	0.766
23					**0.776**	0.739	0.766	0.748	0.751	0.773
24					0.773		0.777	0.751	0.773	0.773
25							0.778		0.774	0.770
26							0.782			**0.782**
27							0.769			**0.781**
28							0.785			**0.779**
29										**0.776**
30										0.772
31										**0.774**
32										0.769
33										0.774

**Table 9:** Performance of MLP classifiers for the intermediate and global rankings of attributes.

Nr	RI_2	RI_3	RI_4	RI_5	RI_6	RI_7	RI_8	RI_9	RI_10	RG
1	0.483	0.483	0.483	0.483	**0.483**	**0.538**	**0.538**	0.483	0.483	0.483
2	0.332	**0.511**	0.511	0.511	**0.511**	0.483	0.484	**0.511**	0.511	0.511
3	0.338	0.292	0.541	**0.541**	**0.483**	**0.534**	0.500	0.331	0.292	0.481
4	0.337	0.488	0.355	0.355	**0.489**	0.485	**0.544**	0.332	0.337	0.535
5	0.482	0.484	0.489	0.489	**0.489**	**0.516**	**0.547**	**0.531**	0.328	0.486
6	0.487	0.407	0.338	0.488	**0.488**	**0.600**	**0.535**	**0.490**	0.392	0.486
7	**0.528**	**0.533**	0.338	0.488	**0.340**	**0.612**	**0.522**	**0.535**	0.196	0.531
8	0.486	0.342	0.453	0.211	**0.487**	**0.614**	**0.510**	0.483	0.649	0.356
9	0.355	0.488	0.538	0.489	0.203	**0.596**	**0.640**	**0.504**	0.616	0.530
10	0.392	0.486	0.493	0.494	**0.487**	0.338	**0.636**	**0.549**	0.492	0.340
11	0.503	0.483	0.404	0.334	**0.489**	0.486	**0.640**	0.487	0.618	0.383
12		0.489	0.261	0.490	**0.341**	0.222	0.203	**0.548**	0.344	0.495
13		0.484	0.488	0.208	**0.232**	0.489	0.472	0.450	**0.693**	0.340
14		0.487	0.349	0.492	**0.434**	0.340	0.495	0.450	0.578	0.489
15		0.495	**0.644**	0.203	**0.531**	0.344	**0.617**	0.343	0.666	**0.606**
16			0.243	0.488	0.354	0.206	0.366	**0.559**	0.564	**0.650**
17			**0.574**	**0.584**	0.203	**0.490**	**0.549**	**0.490**	0.208	0.419
18			**0.581**	0.518	**0.696**	**0.635**	0.494	0.345	0.521	0.493
19			0.349		**0.702**	**0.627**	**0.668**	**0.491**	0.622	0.483
20			0.545		**0.663**	0.420	0.357	0.436	0.373	0.340
21					**0.512**	0.381	0.491	0.344	0.517	0.377
22					**0.690**	**0.511**	**0.648**	**0.491**	0.349	**0.610**
23					**0.684**	0.490	0.295	**0.489**	0.601	**0.684**
24					0.232		**0.605**	0.489	0.464	0.492
25							**0.705**		0.675	**0.616**
26							**0.642**			**0.701**
27							**0.633**			**0.715**
28							0.506			0.344
29										**0.703**
30										0.489
31										**0.690**
32										0.377
33										0.575

**Table 10:** Highest performing feature subsets of intermediate and global rankings.

	RI_2	RI_3	RI_4	RI_5	RI_6	RI_7	RI_8	RI_9	RI_10	RG
**XGBoost**										
acc.	0.645	0.677	0.761	0.729	0.773	0.739	0.785	0.751	0.774	0.774
$n_{k}-j\;feat.$		n−2			n−1	n−4				n−7
max(acc.)		0.678			0.776	0.752				0.782
▵acc.		0.0015			0.003	0.013				0.008
**MLP**										
acc.	0.503	0.495	0.545	0.518	0.232	0.49	0.506	0.489	0.675	0.575
$n_{k}-j\;feat.$	n−4	n−8	n−5	n−1	n−5	n−5	n−3	n−8	n−12	n−6
max(acc.)	0.528	0.533	0.644	0.584	0.702	0.635	0.705	0.56	0.693	0.715
▵acc.	0.025	0.038	0.099	0.066	0.47	0.145	0.2	0.071	0.018	0.14

## Data Availability

Datasets used during the experiments are downloaded from UCI Machine Learning Repository https://archive.ics.uci.edu (accessed on October 2024).
